# Cardiac Sarcoidosis Presented With Hiccups: A Case Report and Literature Review

**DOI:** 10.7759/cureus.40078

**Published:** 2023-06-07

**Authors:** Muhammad Ghallab, Ivan Cancarevic, Nicole C Noff, Daniel Miller, Allison Foster, Zakaria Alagha, Ashraf Sliem, Sanjiv Bakshi

**Affiliations:** 1 Internal Medicine, Icahn School of Medicine at Mount Sinai, New York City (NYC) Health and Hospitals, New York, USA; 2 Internal Medicine, Marshall University Joan C. Edwards School of Medicine, Huntington, USA; 3 Internal Medicine, Flushing Hospital Medical Center, New York, USA; 4 Cardiology, Icahn School of Medicine at Mount Sinai, New York City (NYC) Health and Hospitals, New York, USA

**Keywords:** cardiac imaging, adult cardiac disease, cardiology, hiccups, sarcoidosis

## Abstract

Sarcoidosis is a multisystem disorder of unknown etiology commonly associated with hilar lymphadenopathy and granulomas. Cardiac involvement is less common; however, sarcoidosis is a known cause of restrictive cardiomyopathy. It typically presents as new-onset arrhythmias or heart failure, although cases of sudden cardiac death have been reported. We present a case of a 56-year-old male with a known history of pulmonary sarcoidosis, not on active treatment, who presented to the emergency department with a week of continuous hiccups every few seconds associated with non-exertional dyspnea. An initial computed tomography (CT) scan of the chest showed multiple stellate-like ground-glass opacities and the progression of bronchiectasis. Troponins were negative. On the initial electrocardiogram (EKG), he was found to be in atrial flutter and was admitted to the medical floor. Cardiology was consulted for suspected cardiac sarcoidosis, and they recommended transfer to the tertiary care center for further evaluation.

Upon arrival, the patient underwent catheter ablation for atrial flutter and returned to sinus rhythm after the procedure. The initial nuclear scan with gallium was not suggestive of cardiac sarcoidosis. However, subsequent cardiac magnetic resonance imaging (MRI) showed cardiac involvement.

Due to the high risk of arrhythmias, the patient was scheduled for implantable cardioverter defibrillator placement before discharge. The patient was given oral prednisone. The patient was discharged in stable condition, and interrogation of the device found it well functioning, and no significant arrhythmias were noted.

Presentation of cardiac sarcoidosis can be variable, and any should be considered in any patient with a known history of sarcoidosis who presents with atypical symptoms above the diaphragm, such as hiccups or with new-onset arrhythmias.

## Introduction

Sarcoidosis is an idiopathic, diverse systemic illness that most frequently affects the lungs and lymph nodes or any organ and is characterized by non-necrotizing granulomatous inflammation [1]. About 20-30% of patients with systemic sarcoidosis will have cardiac involvement in the United States, which suggests a worse prognosis. In the United States and Europe, African Americans have a 3.8 times higher frequency of sarcoidosis than Caucasians; while in Japanese patients, cardiac sarcoidosis may be as common as 58% [2,3]. The diagnosis of cardiac sarcoidosis is challenging and can occur alone or in conjunction with multisystem symptoms. The manifestation can range from asymptomatic to high-grade atrioventricular block, ventricular arrhythmia, heart failure, atrial arrhythmias, or sudden cardiac death [1,4]. Hiccups are usually caused by an overly stretched stomach, excessive alcohol drinking or smoking, or rapid emotional changes [5]. Here we present a case of cardiac sarcoidosis that presents with persistent hiccups and then is found to have atrial flutter and newly diagnosed cardiomyopathy.

## Case presentation

**Figure 1:**
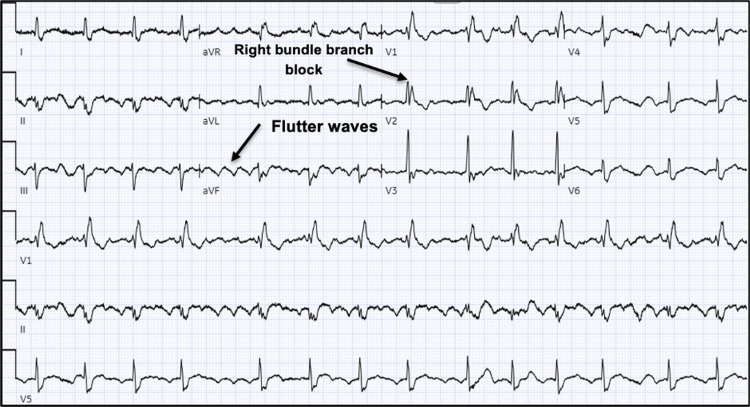
12-leads EKG shows the right bundle branch block and atrial flutter. EKG: electrocardiogram

A 56-year-old male with a past medical history of pulmonary sarcoidosis, sickle cell trait, pre-diabetes, Vitamin D deficiency, and leukopenia presented with ongoing intractable hiccups for one week with associated dyspnea without exertion for one day. The patient was treated for the hiccups three days earlier with chlorpromazine with relief, but ultimately the symptoms returned. The patient was then admitted for chest discomfort as well as abdominal pain. Electrocardiogram (ECG) revealed atrial flutter (Figure 1).

The differential diagnosis list included diaphragmatic irritation from respiratory issues, phrenic nerve injury, and worsening pulmonary sarcoidosis.

**Figure 2:**
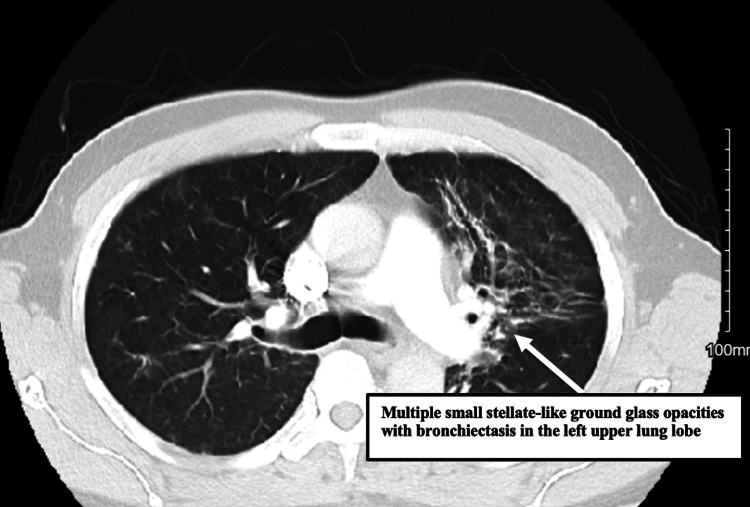
CT pulmonary angiography showing multiple small stellate-like ground glass opacities with bronchiectasis in the left upper lung lobe. CT: computed tomography

A complete blood picture revealed leukopenia and microcytic anemia. The basic metabolic panel and liver function tests were unremarkable. Troponins were negative. Computed tomography angiogram chest with contrast (Figure 2) revealed no pulmonary emboli but did show multiple small stellate-like ground glass opacities with some bronchiectasis.

A transthoracic echocardiogram (Video 1) revealed a reduced left ventricular ejection fraction of 45%, representing newly diagnosed cardiomyopathy, as his baseline ventricular contractility was normal.

**Video 1:** A transthoracic echocardiogram revealing a reduced left ventricular ejection fraction.

**Figure 3:**
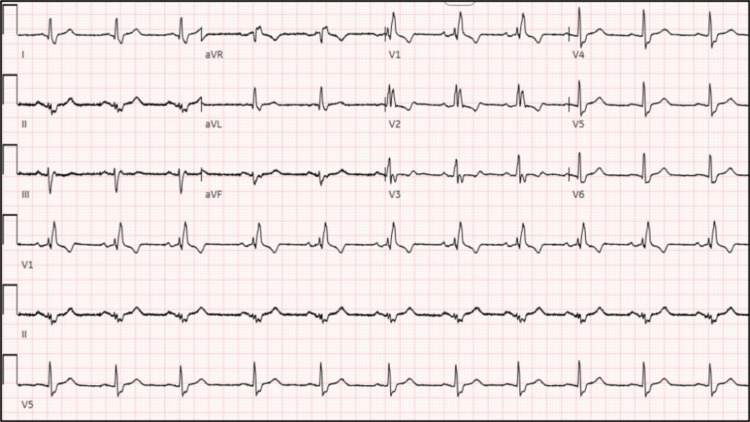
12-leads EKG after the catheter ablation showing right bundle branch block and normal sinus rhythm. EKG: electrocardiogram

The patient had a cardiac ablation of atrial flutter. The ablation eliminated the cavotricuspid isthmus-dependent flutter waves without complications, with a follow-up EKG (Figure 3) showing normal sinus rhythm.

**Figure 4:**
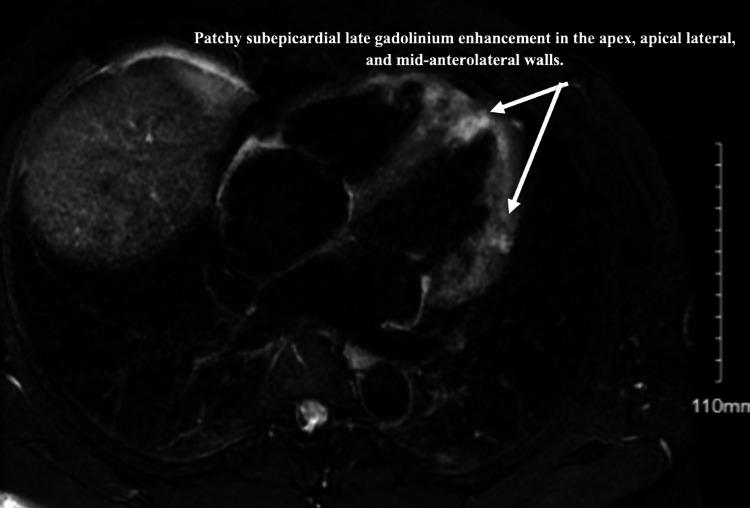
Cardiac MRI - four chambers view showing patchy subepicardial late gadolinium enhancement in the apex, apical lateral, and mid-anterolateral walls. Subendocardial late gadolinium enhancement is evident in the apex as well. MRI: magnetic resonance imaging

**Figure 5:**
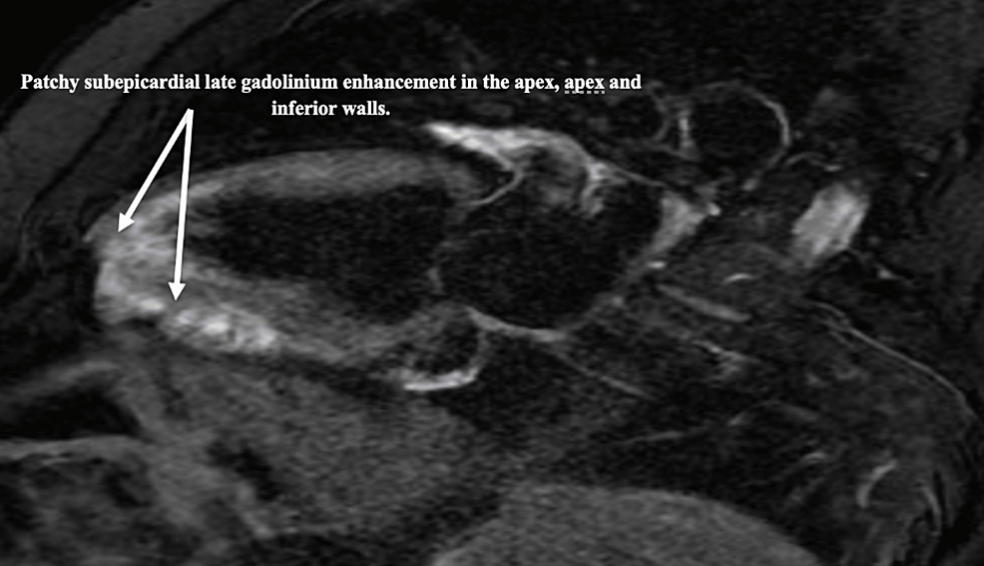
Cardiac MRI - two chambers view showing patchy subepicardial late gadolinium enhancement in the apex and inferior walls. MRI: magnetic resonance imaging

A gallium scan revealed no myocardial uptake. It also showed mild left upper lung lobe uptake, likely due to parenchymal pulmonary sarcoid. Cardiac magnetic resonance imaging (MRI) revealed patchy subepicardial late gadolinium enhancement of the mid-anterolateral wall, apical lateral, inferior walls, and apex, compatible with cardiac sarcoidosis (Figures 4, 5).

Positron emission tomography-computed tomography-fluorodeoxyglucose (PET-CT-FDG) was used to confirm the diagnosis and revealed minimal FDG-avidity in the left ventricular myocardium, suggestive of myocardial inflammation/ sarcoidosis, along with bilateral peri-hilar ground glass and nodularity with FGD-avid hilar foci, likely related to sarcoidosis. The patient then underwent an implantable cardioverter defibrillator (ICD) placement for primary prevention of ventricular arrhythmias and was offered oral prednisone and follow-up in the sarcoidosis clinic.

## Discussion

Sarcoidosis is a rare inflammatory disorder of unknown etiology that includes multiple organ systems, most commonly the lungs, eyes, lymphatic system, skin, and nervous system. However, it less commonly affects the cardiac system, including the atria, ventricles, papillary muscles, pericardium, conduction system, and coronary vessels [6]. Sarcoidosis is most common in young adults, especially African Americans [7]. Sarcoidosis exists in roughly 60 people per 100,000 in the United States, representing 200,000 people currently living with the disease. Of those diagnosed, approximately 5% have cardiac sarcoidosis. However, with advancements in cardiac imaging, cardiac manifestation is likely higher at around 30%.

The most common presentations of cardiac sarcoidosis are atrioventricular block, ventricular dysrhythmias, and heart failure. Less common presentations include bundle branch blocks, atrial dysrhythmias, valvular abnormalities, pericardial effusion, and sudden cardiac death [8]. However, hiccups are among the rarest presentations of sarcoidosis, specifically cardiac sarcoidosis.

A hiccup is an involuntary contraction of the diaphragm and intercostal muscles, causing sudden inspiration and abrupt closure of the glottis. The reflex arc of a hiccup comprises an afferent limb, including the vagus and phrenic nerves, sympathetic chain, central mediator, and an efferent limb, including the phrenic nerve, with accessory efferent neural connections to the glottis and inspiratory intercostal muscles [5]. Possible etiologies of hiccups in patients with sarcoidosis include a lesion along any area of the reflex arc, thoracic lymphadenopathy, and involvement of the central nervous system [5,7].

Treatments for cardiac sarcoidosis involve a multidisciplinary approach. Corticosteroids are the primary medical therapy; however, the precise dosage has yet to be thoroughly studied, given the lack of randomized control trials. The largest cohort study, including 95 Japanese patients treated with a high dose of prednisone (⩾40 mg/d, mean 54 mg/d), was not correlated with increased survival compared to a lower dose (⩽30 mg/d, mean 29 mg/d) [9]. Combining corticosteroids with additional immunosuppression therapies may improve outcomes [8]. A prospective cohort study from Japan, including 17 patients with cardiac sarcoidosis, was split into two groups, either treated exclusively with corticosteroids (n=7) or corticosteroids and low-dose methotrexate (n=10). Combination therapy revealed stable ejection fractions at five years versus the monotherapy group, which developed worsening values [10]. Patients with sarcoidosis and hiccups that were treated with corticosteroids had variable responses. In cases with predominant central nervous system sarcoidosis, more than half responded to steroids or combination therapy [7]. Concomitantly, a high-dose steroid invokes hiccups in its effect on synaptic transmission or neuroexcitatory properties [11]. Alternatively, other pharmacological hiccup treatments can be added concomitantly with sarcoidosis treatment, such as chlorpromazine, dopamine agonists, gabapentin, serotonin, and histamine agonists. Non-pharmacological therapies include phrenic nerve block, vagus nerve stimulator, acupuncture, and breath holding [7].

Nine reported cases of sarcoidosis are presenting with hiccups (Table 1). Of the nine cases, only one had cardiac sarcoidosis. The etiology of the hiccups was either due to central nervous system involvement or mediastinal lymphadenopathy. In five cases, pharmacological treatment failed, and hiccups persisted after corticosteroids were tapered off. Four cases were cured with either only corticosteroid treatment or in addition to clonazepam or methotrexate. In one case, invasive measures were taken and were successful.

**Table 1:** Characteristics of sarcoidosis patients presenting with hiccups.

Case Report	Age, Gender	Cardiac Involvement	Proposed Etiology	Treatment
Neuman et al., 2020 [7]	64, Male	No	Mediastinal lymphadenopathy	Mediastinoscopy and removal of lymph nodes
Kondo et al., 1989 [11]	67, Male	No	Central nervous system	Failed pharmacologic treatment
Chen et al., 2018 [12]	55, Female	Yes	Central nervous system and mediastinal lymphadenopathy	Corticosteroids
Hackworth et al., 2009 [13]	61, Male	No	Mediastinal lymphadenopathy and peritoneal lesions	Failed pharmacologic treatment
Lin et al., 2010 [5]	48, Male	No	Mediastinal lymphadenopathy	Failed pharmacologic treatment
Connolly et al., 1991 [14]	25, Male	No	Central nervous system	Corticosteroids
Douglas et al., 1973 [15]	38, Male	No	Central nervous system	Failed pharmacologic treatment
Miura et al., 2010 [16]	56, Male	No	Central nervous system	Corticosteroids and clonazepam
Seby et al., 2012 [17]	45, Male	No	Central nervous system and mediastinal lymphadenopathy	Corticosteroids and methotrexate

The case presented will be the 10th reported case of sarcoidosis presenting with hiccups and the second with cardiac involvement. The patient was treated with corticosteroids concurrently with metoclopramide with complete resolution of hiccups. Our review of sarcoid patients with hiccups on presentation identifies the common treatments utilized. Although steroids have variable responses, according to the literature, they should still be used first line if there are no contraindications. In conjunction, supporting pharmacologic therapy should be utilized prior to invasive measures. However, even with the treatments available, hiccups may persist long-term.

## Conclusions

Sarcoidosis is a multi-system disease that can affect any organ in the body. We should consider cardiac involvement in patients with a history of sarcoidosis who presents with new cardiac symptoms, including recent onset heart failure, new onset reduction in cardiac contractility, and new onset ventricular and/or supra ventricular arrhythmias. We should also think of other atypical presentations in those patients. Our patient presented with an atypical presentation in the form of hiccups. This led us to further diagnosis of new-onset atrial flutter and new-onset heart failure. Despite the gallium scan being normal, other more sensitive investigations as cardiac MRI and PET-CT confirmed the diagnosis of cardiac sarcoidosis. He underwent ablation of atrial flutter and placement of ICD and was discharged on oral prednisone.
